# PROTOCOL: Co‐Responding Police‐Mental Health Programs and the Impact on Justice and Social Service Outcomes: A Systematic Review

**DOI:** 10.1002/cl2.70051

**Published:** 2025-07-07

**Authors:** Matthew J. Teti, C. Clare Strange, Jordan M. Hyatt, Robert J. Kane

**Affiliations:** ^1^ Department of Criminology and Justice Studies Drexel University Philadelphia Pennsylvania USA

## Abstract

This is the protocol for a Campbell systematic review. The objectives are as follows. The current review will provide criminal justice and policymakers with information regarding the efficacy and effectiveness of co‐responder programs on criminal justice and social service outcomes. Specifically, the authors will address the following research questions: (1) Do co‐responder police‐mental health programs reduce the frequency or likelihood of criminal justice system involvement (e.g., offending, police welfare check, victimization) among those experiencing homelessness, drug and/or alcohol addiction, or mental health crises (hereafter “vulnerable populations”)? (2) Do co‐responder police‐mental health programs improve social service outcomes among vulnerable populations? (3) Do the effects of co‐responder police‐mental health programs vary by the following factors: study research design, geographical location, type of population, type of outcome (e.g., official vs. unofficial reports), and intervention characteristics (e.g., setting, type of practitioners)?

## 1 Background

### 1.1 Problem, Condition, or Issue

Vulnerable individuals, many of whom are experiencing homelessness, drug and alcohol addiction, and/or mental health crises, frequently encounter law enforcement (Lamb and Weinberger 2014; Slate et al. 2013). Correspondingly, these individuals are overrepresented among arrests and police use of fatal force (Ruiz and Miller 2004; de Tribolet‐Hardy et al. 2015). Such overrepresentation in deadly force encounters has led some scholars and policymakers to criticize police training in the area of encounters with persons in crisis in both the United States and abroad (Deane et al. 1999; Kesic et al. 2013; Moore 2010). Historically, these individuals would have been referred to state‐run treatment facilities. However, after the de‐institutionalization of mental health services in the 1960s, law enforcement has become one of the primary first responders for dealing with these individuals in many jurisdictions (Manderscheid et al. 2009; Slate et al. 2013).

This policy shift continues to pose a significant challenge for law enforcement. For example, the number of interactions between police and individuals experiencing mental health issues has increased in recent years (Coleman and Cotton 2016; Shapiro et al. 2015). As a result, there have been a growing number of policy recommendations, programs, and strategies developed to respond to this challenge (Morrissey et al. 2009; Wood and Watson 2017). One commonly suggested, but unevenly implemented, response has been to seek improved partnerships between the criminal justice system and social service systems (Morrissey et al. 2009). The nature of exchanges between law enforcement and vulnerable populations has been undergoing rapid change because of these efforts (Godfredson et al. 2010; Ellis 2014).

The co‐responder model is one example of a partnership approach where police officers and mental health professionals coordinate their response to calls for service related to mental health and other services (Lamb et al. 1995; Morabito and Savage 2021). The co‐responder model has become a commonly employed approach to addressing vulnerable individuals by police agencies in recent years, particularly in the United States, Australia, Canada, and the United Kingdom (Shapiro et al. 2015; Puntis et al. 2018; Robertson et al. 2020). Despite the rising popularity and usage in the field, high‐quality research examining the effectiveness of the co‐responder model is limited (Shapiro et al. 2015; Puntis et al. 2018). These programs are also being developed and deployed (and, to a lesser extent, evaluated) at an increasing rate (Kane et al. 2024; Yang et al. 2024; Bailey et al. 2022; Seo et al. 2021). Therefore, it is necessary to first understand the current landscape of recent co‐responder programs and subsequently examine the impacts that co‐responder models have on criminal justice and social service outcomes among vulnerable populations.

### 1.2 The Intervention

The intervention model being examined in this systematic review comprises various conceptualizations of the police‐mental health co‐responder model. The most salient characteristic of these co‐responder interventions is that, upon a call for service or other interactions, a non‐law enforcement, trained mental health provider (MHP) engages directly with an individual experiencing mental illness or another relevant issue, but there is a law enforcement presence (Morabito and Savage 2021; Shapiro et al. 2015). Service providers in this model can be medical professionals, social workers, peer support specialists, addiction counselors, or other individuals trained to respond to health, social, and addiction issues. The law enforcement presence can be either in‐person or through a cooperation and/or collaboration agreement between the providers. The salient characteristic is that initial, front‐line contact is made by a non‐law enforcement individual, but the service providers actively coordinate their efforts with police and, should an arrest or other formal justice response be needed, police can be summoned or deployed to assume authority over the interaction. Co‐responder models may take a variety of formats (Puntis et al. 2018; White and Weisburd 2017), which is addressed in more detail in the “Criteria for considering studies for this review” section. However, any intervention meeting the above criteria can be included in the current review, including police ride‐alongs, street triage, peer support specialists, remote support, mobile crisis units, plainclothes officers, and uniformed officers paired with mental health clinicians.

### 1.3 How the Intervention Might Work

Although the co‐response model may be implemented in different ways, the goals of each of these permutations are the same: reducing the number of hospitalizations and emergency room admissions of people with mental illness (Meehan et al. 2019; Morabito et al. 2018), while diverting people with mental illness away from criminal justice (in favor of healthcare system) involvement (Lamb et al. 1995; Reuland 2010). Another aim of co‐response is to provide care and treatment to patients whenever and wherever they need it, due to increasing pressures on health systems (Kane et al. 2024). It is through the partnership between police departments and mental health professionals that the various goals of this model are realized.

Crisis intervention teams (CITs) represent what, in healthcare, would be termed a “downstream” treatment, meaning it intervenes at an acute moment in a person's life where the urgency of the encounter often requires split‐second decision‐making (McMahon 2022). Alternatively, “upstream” approaches intervene with an at‐risk person *before* their situation becomes a crisis (McMahon 2022). Treating patients upstream means healthcare teams can take more time with the patient, reduce the risk of verbal and physical conflict, and generally offer a broader set of non‐acute care options (Martins and Burbank 2011). Co‐response policing can be conceptualized as an upstream deployment because it is designed to intercept and treat vulnerable people before their situations – for example, addiction, lack of shelter, and/or emotional/mental well‐being – escalate to crisis levels.

Though not always expressly stated, co‐response policing is rooted in a harm‐reduction conceptual framework designed to minimize potential conflict that might otherwise exist between police and members of vulnerable groups. Harm reduction strategies, emphasizing “pragmatic yet compassionate” care, were developed in Europe as alternatives to the traditional disease‐focused drug addiction interventions (Marlatt 1996). As applied to policing, harm reduction has existed mostly as a proposed philosophy toward the use of police discretion, particularly concerning decreasing discretionary arrests (e.g., Beckett 2016; Kane 2022). A prominent example of this is the Law Enforcement Assisted Diversion (LEAD) program, where police officers divert individuals into treatment rather than arrest; LEAD has become increasingly more prevalent among police departments in the United States (Collins et al. 2019; Perrone et al. 2021). Co‐response policing, which also emphasizes diversion and, to some extent, encourages treatment, can be viewed as a form of harm‐reduction policing, given its alternatives‐to‐enforcement approaches to public behaviors that have long been considered disorderly, such as drug use, sex work, serious mental illness, and homelessness.

**Figure 1:**
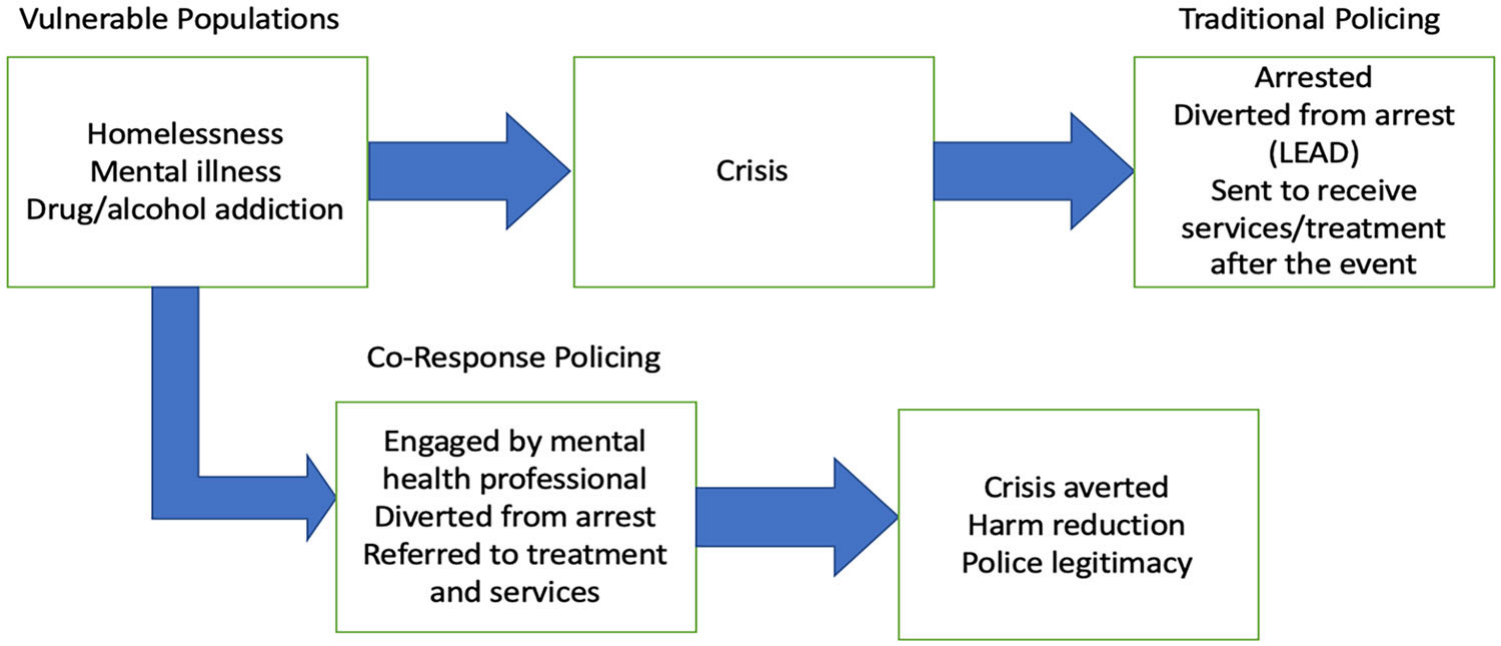
Figure contrasting how the co‐response model and traditional police responses approach vulnerable populations.

Officers learn from the mental health professionals that they are paired with, which increases their confidence and decision‐making skills when dealing with mental health situations (Rosenbaum 2010; Morabito and Savage 2021). Other research has found that citizens who are approached by police officers in conjunction with mental health professionals view the police more favorably, increasing perceptions of legitimacy and procedural justice (Furness et al. 2017; White and Weisburd 2017). The co‐responder model can also increase the willingness of officers to engage with individuals experiencing mental health problems as they are received with more openness and have a greater sense of agency to effectively deal with these issues (White and Weisburd 2017; Bailey et al. 2023). A diagram showing the goals of the co‐response model and how it is supposed to work is shown below in Figure 1.

### 1.4 Why It Is Important to Do This Review

To the current authors' knowledge, there have not been any systematic reviews on police‐mental health co‐responder programs published by the Campbell Collaboration, the Cochrane Collaboration, or other similar organizations. Furthermore, there are no pending reviews currently registered on their respective websites. There have, however, been several reviews of the literature that have been conducted on the topic in recent years in other outlets (Puntis et al. 2018; Dewa et al. 2018; Kane et al. 2018; Parker et al. 2018; Shapiro et al. 2015; Schucan Bird et al. 2017; Eggins et al. 2020; Seo et al. 2021). The present review differentiates itself from these publications and adds to the literature in a number of ways. The reviews conducted by Puntis et al. (2018) and Parker et al. (2018) included qualitative studies and process evaluations, while the reviews done by Dewa et al. (2018), Schucan Bird et al. (2017), and Kane et al. (2018) focus more on CITs and police‐mental health interventions in general. This review exclusively includes quantitative evaluation studies and specifically examines co‐response police‐mental health programs. As such, it will be able to more effectively synthesize the impacts of co‐responder programs on criminal justice outcomes than these other reviews. The 9‐year‐old review conducted by Shapiro et al. (2015) is most like the present review. However, as several studies have been published on co‐responder programs since 2015, it is necessary to provide an updated, more expansive review on the topic. Finally, this review is far more comprehensive than any reviews on the topic published to date because it includes multiple outcomes commonly observed among justice‐involved individuals and potentially a wider range of co‐responder models.

As the number of individuals experiencing homelessness, mental health problems, and substance abuse conditions has increased since the COVID‐19 pandemic, there have been increasingly urgent calls for police agencies to change the ways they interact with these vulnerable populations (Corey et al. 2022; Homelessness Research Institute 2020). Co‐responder models are a relatively new strategy that police agencies are using to respond to mental health calls for service (Morabito and Savage 2021). A rigorous and systematic synthesis of the evidence based on the effectiveness of co‐responder models will allow criminal justice agencies to make informed decisions about policy, practice, and the allocation of resources. A systematic review that examines the impact of co‐response programs on criminal justice and social service outcomes will allow for the identification of common elements and outcomes within these strategies and areas in need of replication. Furthermore, meta‐analytic results can provide new insight into the impact of these models and contribute to the extant evidence base.

## 2 Objectives

The current review will provide criminal justice and policymakers with information regarding the efficacy and effectiveness of co‐responder programs on criminal justice and social service outcomes. Specifically, the authors will address the following research questions.

1. Do co‐responder police‐mental health programs reduce the frequency or likelihood of criminal justice system involvement (e.g., offending, police welfare check, victimization) among those experiencing homelessness, drug and/or alcohol addiction, or mental health crises (hereafter “vulnerable populations”)?
2. Do co‐responder police‐mental health programs improve social service outcomes among vulnerable populations?
3. Do the effects of co‐responder police‐mental health programs vary by the following factors: study research design, geographical location, type of population, type of outcome (e.g., official vs. unofficial reports), and intervention characteristics (e.g., setting, type of practitioners)?

## 3 Methods

### 3.1 Criteria for Considering Studies for This Review

#### 3.1.1 Types of Studies

To be eligible for inclusion in this review, studies are required to use strong quasi‐experimental or randomized experimental designs that test the effects of co‐responder police‐mental health programs on criminal justice and social service outcomes. Due to the difficulty of conducting randomized controlled trials (RCTs) in criminal justice settings, it is necessary to examine quasi‐experimental studies that employ more rigorous design features. Specifically, all quasi‐experimental studies are required to either use a matching procedure when testing differences in the treatment and comparison groups or use statistical controls for baseline group differences. For the purposes of this review, a matched design is one in which there are baseline measures of sociodemographics and/or social service and criminal justice outcomes. Example designs where authors use statistical controls for baseline differences include Bailey et al. (2022), Blais and Brisebois (2021), Yang et al. (2024), and Puntis et al. (2018). Other included designs will be regression discontinuity designs, crossover designs (randomized and non‐randomized), unmatched designs that permit a difference‐in‐difference analysis (e.g., controlled before‐and‐after study), and studies using propensity score matched designs.

Multi‐group comparison studies (including true experimental or quasi‐experimental) where units are allocated to treatment and control groups (through design elements or statistically), will be included in the review. Eligible comparison groups are defined as receiving either no treatment or a traditional law enforcement response (including LEAD), whereby police officers are involved in the initial contacts with vulnerable populations, without any specialized training on how to respond to people experiencing crises.

Relevant existing systematic reviews and/or meta‐analyses will be included for the purposes of harvesting relevant studies that could be eligible for this review. Studies from the published and gray literatures conducted in the English language after 1990 (when the co‐responder model was created) (Morabito and Savage 2021) will be included in the review. There will be no restriction in terms of geographic location; studies can be conducted in any country for the purposes of this review.

#### 3.1.2 Types of Participants

The target population is vulnerable individuals, defined as individuals who are experiencing homelessness (individuals livings on the streets or housing insecure), drug and/or alcohol addiction (individuals that have been diagnosed as a substance use disorder by a mental health professional; or individuals that have shown symptoms of drug/alcohol misuse by way of self‐report instruments or from official data; or individuals that have been labeled as such by the study authors), mental health or another crisis, and/or who have come into contact with law enforcement, either during a traditional law enforcement encounter (e.g., wellness check, pedestrian stop, victimization) or one employing a co‐responder model. Vulnerable individuals across all age ranges (juvenile through adult), races, sex, genders, and ethnicities will be included. A mental health crisis is defined as any situation in which a person's actions, feelings, and behaviors can lead to them hurting themselves or others, and/or put them at risk of being unable to care for themselves or function in the community in a healthy manner (National Alliance on Mental Illness 2023). Studies that include only a subset of the target population (e.g., individuals who are engaged by a co‐responder team that do not fit the description of vulnerable populations) will be excluded.

#### 3.1.3 Types of Interventions

The co‐response model is a deployment (often on foot) that pairs one or more police officers with one or more mental health professionals or social outreach workers while they are out on patrol. These co‐response teams respond to calls for service related to mental health (Lamb et al. 1995; Morabito and Savage 2021) but also broaden their focus to include members of “vulnerable” populations (e.g., people who are addicted, experiencing homelessness, and mental health crisis) (Reuland 2010; White and Weisburd 2017). Studies have reported co‐response models that include police ride‐alongs, remote support, mobile crisis units, plainclothes officers, and uniformed officers (Kisely et al. 2010; Puntis et al. 2018; Thomas and Kesic 2020). The deployment locations and strategies of co‐response models also vary with some being implemented to reactively respond to calls throughout a jurisdiction (Lamanna et al. 2018), some being used to proactively target known crime hot spots (White and Weisburd 2017), and others being constrained and dictated by the implementing police agency's resources (Morabito et al. 2018).

A key difference between models is whether the police and MHP respond to calls for service together or separately. For example, in some models they ride in the same car (Robertson et al. 2020), in others they ride separately but arrive at the same time (Meehan et al. 2019), in others the police call in the MHP only after securing the scene (Yang et al. 2024), and in others the officer consults the MHP by phone while responding to a call (Morabito and Savage 2021). As there is no definitive singular co‐response model, each of these iterations is eligible for inclusion in the review.

#### 3.1.4 Types of Outcomes

The present review focuses on individual‐ and macro‐level criminal justice outcomes as the outcome, operationalized by official indices and self‐reports of arrest, calls for service, citations, victimizations, and use of force incidents. All offense types will be included in this review. Individual‐ and macro‐level social service outcomes, operationalized as number of referrals (e.g., direct connection, warm hand‐off (handoff, i.e., conducted in person, between two members of the health care team), or remote referrals of vulnerable populations to community resources, acceptance of mental health services (both inpatient and community), repeat presentations, and usage of social service agencies are included as the second outcome category. Studies that include only one of the outcomes (criminal justice or social service) or both outcomes will be included in the review. The full coding protocol showing how these outcomes will be classified is shown in Appendix S1A.

### 3.2 Search Methods for Identification of Studies

#### 3.2.1 Electronic Searches

The systematic search will be conducted in the following academic databases:

Australian Criminology Database (CINCH) (Informit).

Criminal Justice Abstracts (EBSCOhost).

PsycINFO (EBSCOhost).

Social Work Abstracts (EBSCOhost).

SocINDEX with Full Text (EBSCOhost).

Cumulative Index to Nursing and Allied Health Literature (CINAHL) (EBSCOhost).

Applied Social Sciences Index & Abstracts (ASSIA) (ProQuest).

Criminal Justice Database (ProQuest).

Dissertations and Theses Global (ProQuest).

International Bibliography of the Social Sciences (IBSS) (ProQuest).

Public Affairs Information Service (ProQuest).

Social Services Abstracts (ProQuest).

Sociological Abstracts (ProQuest).

National Criminal Justice Reference Service (ProQuest.

Conference Proceedings Citation Index – Social Sciences & Humanities (CPCI‐SSH) (Web of Science).

Book Citation Index ‐ Social Sciences & Humanities (Web of Science).

Emerging Sources Citation Index (ESCI) (Web of Science).

Social Science Citation Index (SSCI) (Web of Science).

Campbell Systematic Reviews database (Wiley).

Cochrane Database of Systematic Reviews (Wiley).

Scopus (Elsevier).

Summon (Elsevier).

Medline (National Library of Medicine).

The following open‐access and gray literature sources will be searched:

*Policing*

American Society of Evidence‐Based Policing (https://www.americansebp.org/).

Australian Institute of Criminology (https://www.aic.gov.au/).

Canadian Society of Evidence‐Based Policing (https://www.can-sebp.net/).

Center for Evidence‐Based Crime Policy at George Mason University (https://cebcp.org/).

Center for Problem‐Oriented Policing at Arizona State University (https://popcenter.asu.edu/).

CEPOL (European College of Policing) European Police Science and Research Bulletin (https://www.cepol.europa.eu/scientific-knowledge-and-research/european-law-enforcement-research-bulletin).

College of Policing (UK) (https://www.college.police.uk/).

Danish National Police (Politi) (https://politi.dk/en/).

Finnish Police (Polsi) (https://poliisi.fi/en/frontpage).

Institute for Law and Justice (https://www.ilj.org/index.html).

Justice Research and Statistics Association (https://jirn.org/).

Ministry of Justice (UK) (https://www.gov.uk/government/organisations/ministry-of-justice).

Netherlands Police (Politie) (https://www.politie.nl/en).

New Zealand Police (https://www.police.govt.nz/).

New Zealand Ministry of Justice (https://www.justice.govt.nz/).

Norwegian Ministry of Justice and the Police (https://www.regjeringen.no/en/dep/jd/id463/).

Office of Community Oriented Policing Services (https://cops.usdoj.gov/).

Police Executive Research Forum (https://www.policeforum.org/).

National Policing Institute (https://www.policinginstitute.org/).

RAND Corporation (https://www.rand.org).

Royal Canadian Mounted Police (https://www.rcmp-grc.gc.ca/).

Society of Evidence‐Based Policing (UK) (https://www.sebp.police.uk/).

Swedish National Council on Crime Prevention (Brå) (https://www.government.se/government-agencies/the-swedish-national-council-for-crime-prevention/).

Swedish Police Service (https://polisen.se/en/).

Urban Institute (https://www.urban.org/).

Vera Institute of Justice (https://www.vera.org/).

International Association of Chiefs of Police (https://www.theiacp.org).

CrimeSolutions (https://crimesolutions.ojp.gov).

Canadian Policing Research Catalog (https://www.publicsafety.gc.ca/cnt/cntrng-crm/plcng/cnmcs-plcng/rsrch-prtl/index-en.aspx).

National Research Institute of Police Science (Japan) (https://www.npa.go.jp/nrips/en/).

*Vulnerable Populations*

The National Institute of Mental Health Data Archive (https://nda.nih.gov/).

Mental Health Research Network (https://hcsrn.org/collaboration/cornerstone-projects/mhrn/).

Substance Abuse and Mental Health Data Archive (https://www.datafiles.samhsa.gov/).

National Center for Health Statistics at Center for Disease Control (https://www.cdc.gov/nchs/nhis/index.htm?CDC_AA_refVal=https%3A%2F%2Fwww.cdc.gov%2Fnchs%2Fnhis.htm).

American Psychological Association (https://www.apa.org/research-practice/conduct-research/data-links).

National Institute on Alcohol Abuse and Alcoholism (https://www.niaaa.nih.gov/health-professionals-communities).

U.S. Department of Health and Human Services (https://www.hhs.gov/programs/social-services/homelessness/research/index.html).

National Alliance to End Homelessness (https://endhomelessness.org/ending-homelessness/what-we-do/research/).

National Institute on Drug Abuse (https://nida.nih.gov/research-topics/addiction-science).

Addictions, Drug & Alcohol Institute at University of Washington (https://adai.uw.edu/research/addiction-research-at-the-uw/).

#### 3.2.2 Searching Other Sources

Bibliographies of other relevant reviews (Dewa et al. 2018; Kane et al. 2018; Puntis et al. 2018; Seo et al. 2021; Parker et al. 2018; Kane et al. 2024), and the bibliographies of the included studies themselves, will be consulted to find additional studies to include in the review. To limit publication bias, the authors will also contact the authors of the included studies for any unpublished data or manuscripts, including dissertations and theses. Forward citation searching of included studies will also be conducted using Google Scholar.

#### 3.2.3 Search Terms and Structure

To design the search terms and structure, the authors of the current review harvested search terms from previous studies that have examined the effectiveness of co‐responder police‐mental health programs. This method was adapted from the rigorous strategies often employed in systematic reviews used in the medical field (Varghese and Dugas 2015). First, 10 “gold‐standard” articles were selected from the literature (i.e., studies that best represented the type of studies desired for the current review, in terms of methodology and subject matter). These articles were then entered into PubMed, where a Medical Subject Heading (MeSH) analysis generated a list of common terms across the ten gold‐standard articles (the full MeSH analysis is displayed in Appendix S1B). The authors identified relevant terms from the MeSH analysis and then brainstormed potential variants of each term and Boolean operators (including variants of the terminology, spelling, use of quotations, etc.) to determine the version of each term that would be most effective in retrieving relevant results. Search strings were then created such that studies will retrieved if they contain at least one of the population terms (e.g., “psychiatric,” “mental illness”), at least one of the intervention terms (e.g., “street triage,” “co‐respon*”), and at least one evaluation‐focused terms in either the title, abstract, author‐supplied keywords, or indexing/subject terms. Studies from the published and gray literatures conducted in the English language after 1990 (which is when the co‐responder model was created) will be included in the review. There will be no restriction in terms of geographic location; studies can be conducted in any country for the purposes of this review.

It will be necessary to alter the search string based on databases search functions (e.g., some may not permit searching titles only, or may limit the number of search terms). Any such modifications will be recorded, and all final search strings and their alterations will be reported in the final review for the purposes of study replication. The search string will be entered into each database, and the total number of studies retrieved will be recorded and reported (with duplicates removed). An example of a search conducted in Criminal Justice Abstracts is shown in Table 1 below.

**Table 1:** Example search from Criminal Justice Abstracts.

Line	Syntax
S4	S1 AND S2 AND S3Limiters ‐ Publication Date: 19900101‐20240831; Language: English
S3	TI (effective* OR efficac* OR evaluat* OR experiment* OR interven* OR program* OR quasi‐experiment* OR “quasi experiment*” OR random* OR RCT OR service* OR strategy OR strategies OR train* OR treat* OR trial*) OR AB (effective* OR efficac* OR evaluat* OR experiment* OR interven* OR program* OR quasi‐experiment* OR “quasi experiment*” OR random* OR RCT OR service* OR strategy OR strategies OR train* OR treat* OR trial*) OR KW (effective* OR efficac* OR evaluat* OR experiment* OR interven* OR program* OR quasi‐experiment* OR “quasi experiment*” OR random* OR RCT OR service* OR strategy OR strategies OR train* OR treat* OR trial*) OR SU (effective* OR efficac* OR evaluat* OR experiment* OR interven* OR program* OR quasi‐experiment* OR “quasi experiment*” OR random* OR RCT OR service* OR strategy OR strategies OR train* OR treat* OR trial*)
S2	TI (“co‐respon*” OR “crisis intervention*” OR “crisis team*” OR “emergency respon*” OR “street triage” OR “law enforcement” OR police OR policing) OR AB (“co‐respon*” OR “crisis intervention*” OR “crisis team*” OR “emergency respon*” OR “street triage” OR “law enforcement” OR police OR policing) OR KW (“co‐respon*” OR “crisis intervention*” OR “crisis team*” OR “emergency respon*” OR “street triage” OR “law enforcement” OR police OR policing) OR SU (“co‐respon*” OR “crisis intervention*” OR “crisis team*” OR “emergency respon*” OR “street triage” OR “law enforcement” OR police OR policing)
S1	TI (at‐risk OR “at risk” OR addict* OR crises OR crisis OR distress* OR drug* OR homeless* OR “housing instabil*” OR intoxicat* OR mental* OR overdose* OR psych* OR self‐harm* OR “self‐harm*” OR “sleep* rough” OR “substance use*” OR suicid* OR “wellness check*” OR vulnerable) OR AB (at‐risk OR “at risk” OR addict* OR crises OR crisis OR distress* OR drug* OR homeless* OR “housing instabil*” OR intoxicat* OR mental* OR overdose* OR psych* OR self‐harm* OR “self‐harm*” OR “sleep* rough” OR “substance use*” OR suicid* OR “wellness check*” OR vulnerable) OR KW (at‐risk OR “at risk” OR addict* OR crises OR crisis OR distress* OR drug* OR homeless* OR “housing instabil*” OR intoxicat* OR mental* OR overdose* OR psych* OR self‐harm* OR “self‐harm*” OR “sleep* rough” OR “substance use*” OR suicid* OR “wellness check*” OR vulnerable) OR SU (at‐risk OR “at risk” OR addict* OR crises OR crisis OR distress* OR drug* OR homeless* OR “housing instabil*” OR intoxicat* OR mental* OR overdose* OR psych* OR self‐harm* OR “self‐harm*” OR “sleep* rough” OR “substance use*” OR suicid* OR “wellness check*” OR vulnerable)

## 4 Data Collection and Analysis

### 4.1 Description of Methods Used in Primary Research

Based upon previous reviews on co‐response models, we expect the majority of the evidence found in the search to be quasi‐experimental in nature (Puntis et al. 2018; Dewa et al. 2018; Shapiro et al. 2015; Seo et al. 2021). A model example of a study that evaluates a police co‐responder model was conducted by Morabito et al. (2018). The researchers examined the Boston Emergency Services Team (BEST) of the Boston Medical Center. This partnership resulted in the creation of the BPD's co‐response program, which was launched in January 2011 as a means to team Boston police officers with BEST clinicians. The goal of the program was to provide community‐based psychiatric crisis services to stabilize nonviolent persons experiencing psychiatric emergencies, diverting these individuals from arrest and the criminal justice system when appropriate. Using data from police calls for service, the authors compared various outcomes of mental health‐related incidents conducted by the BEST practitioners to those calls that were not responded to by BEST using a quasi‐experimental design. These outcomes included arrests, drop‐offs at urgent care centers, Boston EMS transport to the emergency department, police transport to the emergency department, individuals being left at the scene, and follow‐ups and referrals to social services.

### 4.2 Criteria for Determination of Independent Findings

There are two main areas that present a risk of nonindependence: single studies having multiple indicators of the same outcome and multiple reports of the same study and that examine the same data (see Higgins et al. 2024). If multiple indicators of an eligible outcome (criminal justice or social service) are reported in a single study, RVE will be used to model all relevant outcomes so that the authors do not choose an outcome themselves. This will ensure as much comparability across studies as possible. If the same data are examined across multiple studies, the authors will use measures from the coding protocol to identify duplicate data (authors' names, time frame, sample, etc.). If multiple publications report results from the same data source, the most complete manuscript using that data source will be used as the primary coding source for the current review. Dependent studies will be grouped together so that single studies can have multiple reports.

### 4.3 Selection of Studies

Study selection will take place in Covidence, following de‐duplication of the search results in EndNote, which is licensed through Drexel University. To select the studies included in the review, the authors will begin by reviewing the titles and abstracts to see if they meet the inclusion criteria – the evaluation of the effectiveness of co‐responder programs on criminal justice or social service outcomes among vulnerable populations. Studies that meet this criteria or studies that cannot be determined from the title or abstract will then have their full text reviewed. The authors of the review will each independently screen half of the studies considered for inclusion at the title and abstract screening stage. It is unlikely that outcomes will be able to be fully determined at this stage; the authors will look to see if the intervention and/or population generally fits the requirements for inclusion in the review at the title and abstract screening stage.

Studies screened as eligible at the title/abstract stage will progress to the full text screening stage. All documents at this stage will be independently double screened based on whether they meet all inclusion criteria (i.e., the intervention is a co‐responder program, outcomes that include criminal justice and/or social service indicators, a sample is considered a vulnerable population).

If the two authors reach different conclusions on whether or not a study should be included in the review at the full‐text screening stage, a third author will make the deciding vote. The number and outcome of these reliability cross‐checks will be recorded and reported. Each author will also independently screen a random sample of 5% of the total studies included in the other screener's group, and reliability measures for agreement will be computed and reported in the final review. Discrepancies will be resolved through a discussion and reaching a consensus between all authors.

### 4.4 Data Extraction and Management

The characteristics of each study's research design, intervention, nature of the outcome measures, and outcome data will be systematically extracted using a coding protocol developed by the authors. The coding protocol examines the study identification, content and methodology, control and treatment sample information, actions received by the control and treatment groups, treatment characteristics, measurement of outcome data, and effect sizes. The coding protocol can be found in Appendix S1A. Coding reliability will be ensured by multiple authors coding each included study (i.e., at least two for any given study). Discrepancies in coding will be brought to a third coder to make the final coding decision.

### 4.5 Assessment of Risk of Bias in Included Studies

Two tools will be used to assess risk‐of‐bias in the included studies in the current review: (a) The Revised Cochrane risk‐of‐bias tool for randomized trials (RoB 2); and (b) The Risk of Bias in Nonrandomized Studies of Interventions (ROBINS‐I) assessment tool.

Two coders from the research team will document the important characteristics of each study using the standard template from the tool. After that, the correct version of the RoB 2 (determined by the type of randomized trial), signaling questions, criteria, response options, and scoring algorithms for each domain the researchers will classify each study as being at “low risk of bias,” “high risk of bias,” or as having “some concerns of bias.” If one domain is coded as having “some concerns of bias” or “high risk of bias,” the study will maintain that overall risk‐of‐bias rating. Each domain will be assessed in every study, even after one domain may be coded as “some concerns of bias” or “high risk of bias.” The domains are biases arising from the randomization process; deviations from the intended interventions; missing or incomplete outcome data; measurement of the outcomes; and the reported results. Domain‐level consensus judgments will be displayed in a table in the main document and the answers and rationale for the signaling questions will be provided in an Appendix.

The ROBINS‐I tool will be used to assess risk‐of‐bias in non‐randomized studies of the effects of interventions. The current review's research questions will be used to identify expected confounders and co‐interventions among the included studies. The ROBINS‐I signaling questions, criteria, response options, and scoring algorithms will be used to assess risk‐of‐bias across seven domains: bias due to confounding; bias in the selection of study participants; bias in classification of the interventions; bias due to deviations from the intended interventions; bias from missing data; bias in the measurement of the outcomes; and bias in the reported results. Each domain will be coded as “low risk of bias,” “moderate risk of bias,” “serious risk of bias,” “critical risk of bias,” and “no information,” with text explaining the justification for the decision. The overall risk level will be based off the “riskiest” domain found in each study. The authors will make efforts to determine the magnitude and potential direction of the biases. The studies' overall strengths and weaknesses will be evaluated to determine the degree to which the intervention effects might be considered causal. Domain‐level consensus judgments will be displayed in a table in the main document, and the answers and rationale behind each signaling question will be provided in an Appendix.

### 4.6 Measures of Treatment Effect

Detailed data will be collected to allow for similar analyses across the studies included in the review. For binary criminal justice outcomes (e.g., arrest, citation, use of force) and social service outcomes (e.g., referrals), odds ratios will be computed, well‐suited for dichotomous outcomes. With continuous measures of these outcomes (e.g., total number of arrests, total number of referrals), a standardized mean difference (SMD) type effect size will be calculated, and an odds ratio created by multiplying the SMD by 1.81, which produces the natural logarithm of the odds ratio (Egli et al. 2009; Lipsey and Wilson 2001). The result will then be exponentiated to produce the odds ratio. Under this strategy, all effect sizes will be in the form of odds ratios for meta‐analysis. For analyses that report “true” rates (number of offenses per person‐year, etc.), incidence rate ratios will be calculated and then compared to odds ratios.

### 4.7 Unit of Analysis Issues

Regarding studies that examine the same outcome at different points in time, the authors will use RVE to model all time‐points used to avoid having to select a specific time‐point and to ensure as much comparability as possible across multiple studies (Lipsey and Landenberger 2006). If a study has more than one comparator and the additional comparators are considered to be eligible for this systematic review by the authors, attempts will be made to combine intervention and comparator conditions so that only a single pairwise comparison is computed, which is recommended by Higgins et al. (2019). This prevents an intervention group from being counted twice and from inflating error related to the unit of analysis Higgins et al. (2019).

Regarding crossover designs, only studies where the authors report the evaluation of carry‐over effects, either through analysis or discussion, will be included. If carry‐over of treatment effects across periods are reported, only results from the first period will be included in the review. If neither carry‐over nor period effects are thought to be a problem, paired t‐tests will be used to compare the outcome measures between treatment periods for each individual participant. The analysis will focus on the within‐participant comparison between treatment arms rather than treating each treatment period as a separate unit.

If studies include multiple treatment arms, the authors will combine eligible multiple treatment versus comparator conditions so that there is only one pairwise comparison made, as detailed below:

1. Synthesis for co‐response program v no treatment.
2. Synthesis for co‐response program v traditional law enforcement response.

### 4.8 Dealing With Missing Data

While the criteria for inclusion in the review have been outlined previously in the protocol (i.e., the intervention is a co‐responder program, outcomes that include criminal justice and/or social service indicators, and a sample is considered a vulnerable population), the integrity of the study itself must also be taken into account. Missing data from the original studies included in the current review can override decisions made regarding study eligibility and inclusion, as well as effect size calculation; that is, a study could fit all of the criteria for inclusion but still be excluded from the review due to missing data. Therefore, the authors will attempt to obtain necessary relevant data from the authors of the original studies. If unsuccessful, the researchers will include the studies in the summary section of the report in a narrative form, along with an explanation as to why they are not present in the meta‐analysis.

### 4.9 Assessment of Heterogeneity

A *χ* ^2^ test, specifically Cochran's *Q* test, where a significant chi‐square value indicates the presence of substantial variation between study results beyond what would be expected by chance alone, will be used to assess heterogeneity of effect sizes in the current review. A *p*‐value of 0.05 will be used as the cutoff, as is standard practice. Consistent with Cochrane Collaboration guidelines, the authors also calculate the *I* ^2^ statistic, which is used to measure heterogeneity in effect sizes (Deeks et al. 2019). Between‐study variance (tau‐squared) will be reported as well.

### 4.10 Assessment of Reporting Biases

To test reporting biases, the authors will conduct a PET‐PEESE model as a regression to correct for the correlation between effect sizes and standard errors or effect sizes and standard errors squared (Stanley and Doucouliagos 2014).

### 4.11 Data Synthesis

The current review will follow the standards of meta‐analysis described in Practical Meta‐Analysis by Lipsey and Wilson (2001) and will use the metafor package in R and Stata for the meta‐analyses; (Viechtbauer 2010) RVE will be run in R using the clubSandwich package (Pustejovsky 2022). RCTs and quasi‐experiments will be meta‐analyzed together. To compute mean‐effect sizes, the inverse variance weight method of the meta‐analysis will be used (Lipsey and Wilson 2001). The results of the meta‐analyses will be displayed using tables and forest plots and will be grouped according to outcome (or category, see above) and risk of bias. Each estimate will be presented with its associated 95% confidence interval.

In terms of outcomes, criminal justice involvement may include studies that measure official indices and self‐reports of arrest, calls for service, citations, victimization, and use of force incidents. Social service involvement may include remote or warm handoffs or referrals to social service agencies, which will also be combined into one conceptual grouping for meta‐analysis if needed. If there is only one effect size for an outcome category, a standardized effect size will be presented. If there are two or more studies with similar outcomes (referrals and warm handoffs) then all outcome types will be combined using RVE with dummy coding so that an estimate can be provided for each outcome type (Park and Beretvas 2018).

### 4.12 Subgroup Analysis and Investigation of Heterogeneity

In addition to examining the impact of co‐responder programs on criminal justice and social service outcomes, one of the major objectives of this review is to examine the effects of different forms of co‐response. To that end, the authors will conduct moderator analyses using meta regression techniques (e.g., Deeks et al. 2019) to examine effects across specific subgroups based on characteristics of study research design, geographical location, type of population, type of outcome (e.g., official vs. unofficial reports), and intervention characteristics (e.g., setting, type of practitioners, ride‐along vs. mobile support, etc.).

### 4.13 Sensitivity Analysis

Sensitivity analyses will be conducted to test the robustness of the results, including examining the impact of risk of bias and adjusting for different intraclass correlation values that could impact effect estimates. The authors will use meta‐regression to analyze the differences between small and large sample sizes (Deeks et al. 2019). Should meta‐regression not be possible, the one‐study‐removed approach will be employed.

### 4.14 Treatment of Qualitative Research

Qualitative research will not be included in the review.

## Author Contributions

Content: Matthew J. Teti, C. Clare Strange, Jordan M. Hyatt, and Robert J. Kane. Systematic review methods: Matthew J. Teti, C. Clare Strange, and Jordan M. Hyatt. Statistical analysis: Matthew J. Teti. Information retrieval: Matthew J. Teti, C. Clare Strange, and Robert J. Kane.

## Conflicts of Interest

The authors declare no conflicts of interest.

## Supporting information

Appendix_A_Co_Responder_Protocol_FINAL.

Appendix_B_Co_Responder_Protocol_FINAL.
