# Supplementary material for: PROTOCOL: Co‐Responding Police‐Mental Health Programs and the Impact on Justice and Social Service Outcomes: A Systematic Review
Source: Campbell Syst Rev. 2025 Jul 7;21(3):e70051. doi: 10.1002/cl2.70051 (PMC12230866; doi:10.1002/cl2.70051)
Supplement: Supplementary file 1 — Appendix_A_Co_Responder_Protocol_FINAL. [file CL2-21-e70051-s001.docx]

**APPENDIX A: CODING PROTOCOL**

1. **STUDY IDENTIFICATION**

**Identification number: [studyID] __**

**Author: [author] __**

**Name of funding agent: [fund] __**

**Title: [title] __**

**Other publication about this study: [crossref1] __**

**Other publication about this study: [crossref2] __**

**Other publication about this study: [crossref3] __**

**Publication year: [pubyear] __**

**Geographic location of study: [location] __**

**Publication type: [pubtype] __**

**1** – Book

**2** – Book chapter

**3** – Federal report

**4** – State or local report

**5** – Journal

**6** – Non-published (grey literature)

**Is the assessment of the effects of treatment on criminal justice outcomes included in this study: (yes==1; no==0) [crim]**

**Is the assessment of the effects of treatment on social service outcomes included in this study: (yes==1; no==0) [service]**

**Is the study limited to vulnerable populations: (yes==1; no==0) [vulnpop]**

****** If a single study is reported in multiple documents, the study identification number is the primary publication. Any further document will be listed under “*Other publication about this study”.*

1. **ELGIBILITY CRITERIA**
2. **Content**

To be eligible a study must meet one of the following criteria. Answer each question with a “yes” or “no.”

YES NO

The study evaluates the effects of a co-responder police mental health program on criminal justice outcomes (e.g. arrest, criminal and non-criminal citations, and use of force incidents). The measure may be based on official records or self‐ report and may be reported on a dichotomous or continuous scale among vulnerable populations.

The study evaluates the effects of a co-responder police mental health program on the social service outcomes (e.g. referrals to treatment services). The measure may be based on official records or self‐ report and may be reported on a dichotomous or continuous scale among vulnerable populations.

1. **Assignment to Groups:**

**Inclusion:** only studies meeting criteria a, b, c, or d will be included YES

- 1. Randomized
  2. Quasi-randomized
  3. Matched groups
  4. Use of control variables to account for initial group differences which go beyond gender, race/ethnicity, and age
  5. Use of control variables limited to gender, race/ethnicity, and age
  6. No use of control variables

1. **PROGRAM DESCRIPTION**

**A – What happens to the control group? [contrgrp] __**

**1 –** No treatment

**2 –** Traditional law enforcement response

**3 –** Other____________

**4 –** Cannot tell

**B – What happens to the treatment group? [treatgrp] __**

**1 –** Police ride along

**2 –** Peer support specialists

**3 –** Remote support

**4 –** Mobile crisis units

**5 –** Uniformed officers paired with mental health clinicians

**C – Rating of initial group** **similarity: [simRate] __**

**(7 = highly similar; 1 = highly dissimilar)**

7 **–** Randomized design, large *N* or small *N* with matching;

5 **–** Nonrandomized design with strong evidence of initial equivalence;

1 **–** Nonrandomized design, comparison group highly likely to be different from treatment group

**D – Attrition problems present: (yes==1; no==0) [attrit] __**

**E – Use of statistical significance test: (yes==1; no==0) [SigTest] __**

1. **METHODOLOGICAL RIGOR ASSESSMENT**
2. **Used control variables in statistical analysis to account for initial group differences (yes = 1; no=0) [cntrvar] __**
3. **Used subject-level matching (yes = 1; no=0) [matching] __**
4. **Variables used to control/match on pre-test differences YES**
   1. Age
   2. Gender
   3. Race
   4. Ethnic background or national origin
   5. Housing status
   6. Employment status
   7. Other
5. **Variables used for statistical control YES**
   1. Age
   2. Gender
   3. Race
   4. Ethnic background or national origin
   5. Housing status
   6. Employment status
   7. Other
6. **Rating of initial group similarity: [simRate] __**

**(7 = highly similar; 1 = highly dissimilar)**

7 **–** Randomized design, large *N* or small *N* with matching;

5 **–** Nonrandomized design with strong evidence of initial equivalence;

1 **–** Nonrandomized design, comparison group highly likely to be different from treatment group

1. **Attrition problems present: (yes==1; no==0) [attrit] __**
2. **Use of statistical significance test: (yes==1; no==0) [SigTest] __**
3. **SIZE AND COMPOSITION OF THE SAMPLE**

**A – Size**

Total *N* in treatment group **[size1] __**

Total *N* in control group **[size2] __**

**B – Composition**

**Information available: (yes==1; no==0; partially==2) [Comp1] __**

**Treatment Group:**

Age: (mean) **[age] __**

Gender: male (%) **[gender] __**

Employment status: unemployment (%) **[empl] __**

Race differentiated: (yes==1; no==0) **[race0] __**

White (%) **[race1] __**

Black (%) **[race2] __**

Asian (%) **[race3] __**

Latino (%) **[race4] __**

Others (specify): (%) **[race5] __**

National origin differentiated: (yes==1; no==0) **[origin0] __**

Nonimmigrant **[origin1] __**

Immigrant **[origin2] __**

Housing status: homelessness (%) **[homeless] __**

**Control Group:**

Age: (mean) **[age] __**

Gender: male (%) **[gender] __**

Employment status: unemployment (%) **[empl] __**

Race differentiated: (yes==1; no==0) **[race0] __**

White (%) **[race1] __**

Black (%) **[race2] __**

Asian (%) **[race3] __**

Latino (%) **[race4] __**

Others (specify): (%) **[race5] __**

National origin differentiated: (yes==1; no==0) **[origin0] __**

Nonimmigrant **[origin1] __**

Immigrant **[origin2] __**

Housing status: homelessness (%) **[homeless] __**

**Eventual additional treatment or comparison groups (2 and following).**

**Add additional pages.**

Age: (mean) **[age] __**

Gender: male (%) **[gender] __**

Employment status: unemployment (%) **[empl] __**

Race differentiated: (yes==1; no==0) **[race0] __**

White (%) **[race1] __**

Black (%) **[race2] __**

Asian (%) **[race3] __**

Latino (%) **[race4] __**

Others (specify): (%) **[race5] __**

National origin differentiated: (yes==1; no==0) **[origin0] __**

Nonimmigrant **[origin1] __**

Immigrant **[origin2] __**

Housing status: homelessness (%) **[homeless] __**

1. **OUTCOME INFORMATION**

**A – Criminal justice construct represented by this measure: (yes==1; no==0)**

1. Arrest **[mea1] __**
2. Criminal citation **[mea2] __**
3. Use of force incident **[mea3] __**
4. Call for service **[mea4] __**
5. Victimization **[mea5] ___**
6. Other (specify:) **[mea6] __**

**B – Social service construct represented by this measure: (yes==1; no==0)**

1. Direct referral **[mea6] __**
2. Physical connection/warm handoff **[mea7] __**
3. Remote referral **[mea8] __**
4. Other (specify:) **[mea9] __**

**C – Type of measurement scale: (yes==1; no==0)**

1. Dichotomy **[scale1] __**
2. Trichotomy **[scale2] __**
3. 4 or more discrete ordinal categories **[scale3] __**
4. Continuous **[scale4] __**
5. Survival measure **[scale5] __**

**D – Source of data: (yes==1; no==0)**

1. Self-report **[source1] __**
2. Police official records **[source2] __**
3. Other (specify) **[source3] __**
4. Cannot tell **[source4] __**

**E – Are the measures valid? [valid] __**

**(1==questionable; 2==acceptable)**

**7. EFFECT SIZE INFORMATION**

**A – Treatment group identifier [ES_gro1] __**

**B – Control group identifier [ES_gro2] __**

**C – Effect size identifier [ES_ident] __**

(Number each effect size within a study sequentially)

**D – Effect size type: [ES_type] __**

**1 –** Baseline (pre-test; prior to the start of the intervention)

**2 –** Post-test (first measurement point, post intervention)

**3 –** Follow-up (all subsequent measurement points, post intervention)

**E – Criminal justice outcome indicator: [ES_cjind] __**

**1 –** Arrest

**2 –** Citation

**3 –** Use of force incident

**4 –** Call for service

**5** - Victimization

**6 –** Other (specify)

**F – Social service outcome indicator: [ES_ssind] __**

**1 –** Direct referral

**2 –** Physical connection/warm handoff

**3 –** Remote referral

**4 –** Other (specify):

**G – Measurement type:**

**1 –** Proportion **[ES_meast1] __**

**2 –** Mean frequency **[ES_meast2] __**

**3 –** Survival frequencies **[ES_meast3] __**

**H – Time frame in months captured by measure**

**1 –** Minimum **[ES_len1] __**

**2 –** Maximum **[ES_len2] __**

**3 –** Mean **[ES_len3] __**

**4 –** Fixed (same for all subjects) **[ES_len4] __**

**8. EFFECT SIZE DATA**

**A – Sample size**

**1 –** Treatment group **[ES_sam1] __**

**2 –** Control group **[ES_sam2] __**

**B – Means and standard deviation**

**1 –** Treatment group mean **[ES_mean1] __**

**2 –** Control group mean **[ES_mean2] __**

**3 –** Treatment group standard deviation **[ES_stdev1] __**

**4 –** Control group standard deviation **[ES_stdev2] __**

**C – Proportion**

**1 –** Proportion of treatment group that received criminal justice outcomes **[ES_prop1] __**

**2 -** Proportion of control group that received criminal justice outcomes **[ES_prop2] __**

**3 –** Proportion of treatment group that received social service outcomes **[ES_prop3] __**

**4 -** Proportion of control group that received social service outcomes **[ES_prop4] __**

**D – Survival**

**1 –** Mean survival time of the treatment group **[ES_surv1] __**

**2 –** Mean survival time of the control group **[ES_surv2] __**

**E – Odds ratio (logistic regression) not having CJ outcome/having CJ outcome**

**1 –** Calculated for the treatment group **[ES_odds1] __**

**2 –** Calculated for the control group **[ES_odds2] __**

**F – Which group does the raw effect favor (ignoring statistical significance)?**

**1 –** Treatment group **[ES_eff] __**

**2 –** Control group

**3 –** Neither (effect size equals zero)

**4 –** Cannot tell (effect size cannot be used if this option is selected)

**G – Is this difference reported as statistically significant?**

**0 –** No **[ES_sig] __**

**1 –** Yes

**2 –** Not tested

**3 –** Cannot tell

**H – Type of statistical test**

**1 –** T-test **[ES_test] __**

**2 –** F-test

**3 –** Chi-square

**4 –** Regression analysis, including logistic regression

**5 –** Other

**8 –** No analysis/NA

**9 –** Cannot tell
