# Supplementary material for: PROTOCOL: Co‐Responding Police‐Mental Health Programs and the Impact on Justice and Social Service Outcomes: A Systematic Review
Source: Campbell Syst Rev. 2025 Jul 7;21(3):e70051. doi: 10.1002/cl2.70051 (PMC12230866; doi:10.1002/cl2.70051)
Supplement: Supplementary file 2 — Appendix_B_Co_Responder_Protocol_FINAL. [file CL2-21-e70051-s002.docx]

**APPENDIX B: MeSH ANALYSIS RESULTS**

| **PMID** | 29920560 | 29052275 | 35103364 | 29588323 | 30111302 | 25239523 | None | None | None | None |
| --- | --- | --- | --- | --- | --- | --- | --- | --- | --- | --- |
| **Title** | Evidence for the effectiveness of police-based pre-booking dive... | Effectiveness of current policing-related mental health interve... | Re-examining mental health crisis intervention: A rapid review ... | Interagency collaboration models for people with mental ill hea... | A systematic review of co-responder models of police mental hea... | Co-responding Police-Mental Health Programs: A Review. | Examining proactive and responsive outcomes of a dedicated co-res… | Police response to people with mental illnesses in a… | Police pre-arrest diversion of people with mental health issues: A syste... | A co-responder model for policing mental health problems at crime hot spots: Find… |
| **Author Year** | Dewa CS (2018) | Kane E (2018) | Marcus N (2022) | Parker A (2018) | Puntis S (2018) | Shapiro GK (2015) | Morabito MS (2021) | Morabito MS (2018) | Shucan Bird KL (2017) | White C (2017) |
| **MeSH Headings** |  | Crisis Intervention | Crisis Intervention / methods | Cooperative Behavior* | Criminal Behavior* Criminal Law / methods Criminal Law / standards | Community Mental Health Services Community-Institutional Relations* Crisis Intervention* |  |  |  |  |
|  |  |  |  |  |  | Emergency Services, Psychiatric* |  |  |  |  |
|  | Female |  |  |  |  |  |  |  |  |  |
|  | Hospitalization Humans | Humans | Humans | Health Personnel* Humans | Humans | Health Services Research* Humans |  |  |  |  |
|  |  |  |  | Law Enforcement* | Law Enforcement / methods |  |  |  |  |  |
|  | MEDLINE Male Mental Disorders / epidemiology* Mental Disorders / physiopathology | Mental Health / standards* Mental Health Services / standards* | Mental Disorders* / psychology Mental Disorders* / therapy Mental Health Mental Health Services* | Mental Disorders* Mental Health Services* Mental Health* | Mental Disorders / psychology* Mental Disorders / therapy* Mental Health Services / standards Mental Health* / standards |  |  |  |  |  |
|  | Police / legislation & jurisprudence* | Police / standards* | Police | Police* | Police / psychology* Police / standards | Police* Program Evaluation |  |  |  |  |
|  |  |  | Qualitative Research |  |  |  |  |  |  |  |
|  |  |  |  |  | Triage / methods* Triage / standards |  |  |  |  |  |
| **Author Assigned Keywords** |  |  | community mental health services crisis intervention emergency services mental health police psychiatric | interagency collaboration mental health police systematic scoping review | Crisis team Mental health crisis Police and mental health Street triage |  |  |  |  |  |
